# Pegylated gold nanoparticles interact with lipid bilayer and human serum albumin and transferrin

**DOI:** 10.1038/s41598-024-74898-0

**Published:** 2024-10-18

**Authors:** Elżbieta Okła, Sylwia Michlewska, Adam Buczkowski, Serafin Zawadzki, Katarzyna Miłowska, Javier Sánchez-Nieves, Rafael Gómez, Francisco Javier de la Mata, Maria Bryszewska, Janusz Blasiak, Maksim Ionov

**Affiliations:** 1 Faculty of Biology and Environmental Protection, Department of General Biophysics, University of Lodz, Pomorska 141/143, Lodz, 90–236 Poland; 2 University of Lodz Doctoral School of Exact and Natural Sciences, 21/23 Matejki St., Lodz, 90-237 Poland; 3 Faculty of Biology and Environmental Protection, Laboratory of Microscopic Imaging and Specialized Biological Techniques, University of Lodz, Banacha 12/16, Lodz, 90–237 Poland; 4 Faculty of Chemistry, Department of Physical Chemistry, Division of Biophysical Chemistry, University of Lodz, Pomorska 165, Lodz, 90-236 Poland; 5 BioMedChem Doctoral School of the University of Lodz and Lodz Institutes of the Polish Academy of Sciences, 21/23 Matejki St., 90‑237 Lodz, Poland; 6 Universidad de Alcalá Department of Organic and Inorganic Chemistry, and Research Institute in Chemistry “Andrés M. del Río” (IQAR), Spain and Instituto Ramon y Cajal de Investigacion Sanitaria, IRYCIS, Colmenar Viejo Road, Km 9, 100, Madrid, 28034 Spain; 7 Networking Research Center on Bioengineering, Biomaterials and Nanomedicine (CIBER-BBN), Madrid, Spain; 8 Collegium Medicum, Faculty of Medicine, Mazovian Academy in Plock, Pl. Dabrowskiego 2, Plock, 09-402 Poland

**Keywords:** Pegylated gold nanoparticles, Serum human albumin, Transferrin, Protein corona, DMPC lipid membranes, Liposomes, Biophysical chemistry, Membrane biophysics, Nanoscale biophysics, Cell biology, Drug discovery

## Abstract

Gold nanoparticles (AuNPs) are potentially applicable in drug/nucleic acid delivery systems. Low toxicity, high stability, and bioavailability are crucial for the therapeutic use of AuNPs and they are mainly determined by their interactions with proteins and lipids on their route to the target cells. In this work, we investigated the interaction of two pegylated gold nanoparticles, AuNP14a and AuNP14b, with human serum proteins albumin (HSA) and transferrin (Tf) as well as dimyristoyl-phosphatidylcholine (DMPC) liposomes, which can be a representative of biomembranes. We showed that AuNP14a/b interacted with HSA and Tf changing their electrical, thermodynamic, and structural properties as evidenced by dynamic light scattering, zeta potential, transmission electron microscopy, circular dichroism, fluorescence quenching, and isothermal titration calorimetry. These nanoparticles penetrated the DMPC membrane suggesting their ability to reach a target inside the cell. In most of the effects, AuNP14b was more effective than AuNP14a, which might result from its more positive charge. Further studies are needed to evaluate whether the interaction of AuNP14a/b with HSA and Tf is safe for the cell/organism and whether they may safely penetrate natural membranes.

**Supplementary Information:**

The online version contains supplementary material available at 10.1038/s41598-024-74898-0.

## Introduction

Colloidal gold nanoparticles (AuNPs) are considered non-toxic therapeutic drug/nucleic acid carriers and therefore it is important to assess their interaction with biomolecules that can be encountered on their way to the target site^1,2^. In a biological environment, AuNPs can induce a cascade of different mechanisms and interactions, depending on their size, shape, charge, and cargo Xu^3^. In this work, we investigated two pegylated gold nanoparticles, AuNP14a and AuNP14b, with the dendron: polyethylene glycol (PEG) ratio 3:1 and 1:1, respectively, and their interaction with human serum albumin (HSA), transferrin (Tf) and lipid membranes that may impede the transit of nanoparticles and induce unwanted side effects. The synthesis, biophysical properties, and cytotoxic effects of these AuNPs were shown in our previous works^4–7^. We showed that those AuNPs could be considered carriers of siApoE4 (anti-ApoE4 siRNA), a siRNA targeting a major genetic risk factor for Alzheimer disease^7,8^.

We chose HSA as it is the major plasma protein, may effectively bind diverse ligands and so is widely used in biological studies of nanomaterials^9–11^. Transferrin is a blood glycoprotein essential in iron transport and the maintenance of the blood-brain barrier (BBB), and therefore involved in the pathogenesis of many human disorders^12,13^. Therefore, disturbance in the structure and functions of these proteins by nanoparticles may lead to their toxic effects.

The small size and high surface-to-volume ratio of nanoparticles facilitate their binding to biomolecules and the formation of “protein corona”^10,11,14^. Covering NPs with proteins depends on the characteristics of nanomaterial (shape, size, curvature, surface area) and is driven by various forces, including Van der Waals interactions, hydrogen bonds, or electrostatic interactions^15^. However, some NPs can inhibit protein secondary structure conversion and consequently the inflammatory reaction^16^. It was shown that pre-coating of NPs by proteins prevented their removal by the immune system before cellular uptake and facilitated reaching the target site^17^. This phenomenon occurs in the case of transferrin-coated nanoparticles^18^. Since Tf receptors are present in the BBB cells, Tf is a common choice of protein corona to improve drug delivery to the brain^18–20^.

Nanoparticles can also interact with biomembranes containing lipid bilayers with hydrophilic and hydrophobic parts essential for the uptake of nanoparticles^21^. Cationic AuNPs can penetrate the lipid bilayer through the hydrophobic tail region^22^. However, in vivo NPs interact with membranes as a nanoparticle-protein agglomerate. This can decrease the adhesion of NPs to the cell membrane and their cytotoxic effect^23,24^. The modification of lipid structure after interaction with nanoparticles depends on the charge of NPs but also its other physicochemical features, including size and shape as well as membrane complexity and its surface tension^25^. Moreover, the adhesion forces alter the elastic lipid membrane, so it can wrap around a nanoparticle. The energy of adhesion determines whether the nanoparticle will be wrapped partly or wholly and consequently, it will be internalized and anchored in the lipid membrane or completely engulfed through endocytosis^26^. Therefore, studying the nanoparticle–membrane interaction is important in the understanding of NPs bioactivity and biodistribution.

In this work, we investigated the interaction of AuNP14a/b with HSA, Tf, and lipid membranes using dynamic light scattering, zeta potential, transmission electron microscopy, circular dichroism, fluorescence quenching, and isothermal titration calorimetry.

## Materials and methods

### Materials

**Figure 1:**
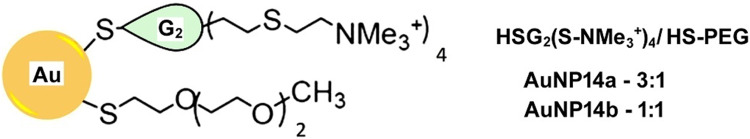
Structure of pegylated gold nanoparticles of second generation (G_2_) with carbosilane dendron (left panel). The cationic dendron, HSG2(SNMe^3+^)_4_ and commercial PEG ligand CH_3_O(CH_2_CH_2_O)nCH_2_CH_2_SH, HS-PEG with the presentation of dendron/PEG ratio (right panel).

Two pegylated gold nanoparticles, AuNP14a and AuNP14b, with carbosilane dendrons were synthesized at the University of Alcala (Alcala de Henares, Spain) as described previously [5]. AuNP14a and AuNP14b have the same gold core and the dendron to PEG ratio 3:1 and 1:1, respectively (Fig. 1). Either AuNP was taken from its stock (1 mg/mL) phosphate buffer solution, pH 7.4, and added to the working sample to give a final concentration in the range of 5–90 µg/mL. Control samples received buffer only. Human serum albumin was purchased from Sigma Aldrich (St. Louis, MO, USA) and human transferrin from Biorbyt Ltd (Cambridge, UK). 1,2-dimyristoyl-sn-glycero-3-phosphocholine (DMPC) was obtained from Fluka (Buchs, Switzerland).

### Zeta potential and dynamic light scattering

Zeta potential and hydrodynamic diameter of proteins titrated with AuNP14a/b (5–60 µg/mL) were measured in phosphate buffer (10 mM, pH7.4) on Zetasizer Nano ZS analyzer (Malvern Instrument Ltd., Malver, UK). The concentration of proteins was 0.25 µM. The results of zeta potential were calculated with the Helmholtz–Smoluchowski equation. Results were collected from 3 measurements, 7 runs each. Dynamic light scattering (DLS) measurements provided information about the size of proteins and agglomerates they formed with AuNPs. The backscatter was set for 173°, λ_abs_ 720 nm and refractive index (RI) was 0.28. The particle size was determined from 3 independent replicates- the average of 5 cycles each.

### Circular dichroism

Circular dichroism (CD) measurements were performed to analyze changes in the secondary structure of HSA and Tf in the presence of AuNPs. Stock solutions of proteins at a final concentration of 0.5 µM each were prepared in the phosphate buffer and measured on a Jasco J-815 CD spectrometer (Jasco International Co., Ltd., Tokyo, Japan) at a wavelength set from 195 to 260 nm using a 0.5 nm quartz cell.

### Fluorescence measurements

All fluorescence measurements were performed with protein solutions at 4 µM on a PerkinElmer LS-50B spectrofluorometer (PerkinElmer, Inc., Waltham, MA, USA). Quenching of fluorescence emitted by tryptophan of HSA and Tf upon AuNPs addition was observed at the wavelength emission range 305–445 nm (λ_exc_ 295 nm). The monochromator slits were set at 2.5 nm for excitation and 10 nm for emission. To assess the interaction of AuNPs with DMPC liposomes. Two fluorescent probes: DPH (1,6-diphenylhexatriene) and its hydrophilic derivative TMA-DPH (1-(4-Trimethylammoniophenyl)-6-phenyl-1,3,5-hexatriene p-toluenesulfonate) (Sigma Aldrich, St. Louis, MO, USA) were separately used to determine whether the gold nanoparticles penetrate hydrophobic or hydrophilic region of the liposomal lipid bilayer, respectively. Samples were prepared in phosphate buffer. Both fluorescent probes were used at 2 µM, and DMPC was at 30 µg/mL. The excitation/emission wavelengths for DPH and TMA-DPH were 348/358 nm and 426/428 nm, respectively. An appropriate fluorescent probe was mixed with DMPC liposomes and AuNPs before measurements.

### Transmission electron microscopy

Transmission electron microscope JEOL-1010 (JEOL, Tokyo, Japan) was used to take a picture of protein corona forming around AuNPs and to show interactions between tested nanoparticles (AuNP14a, AuNP14b) and DMPC liposomes. Samples were prepared in the phosphate buffer and incubated (15 min, 37 °C) before placing them on a copper grid with a carbon surface. Grids with samples were stained with uranyl acetate solution for 2 min, washed, and dried at room temperature for 5 min. Then samples were examined in a transmission electron microscope.

### Liposomes preparation

10 mg DMPC was dissolved in 400 µL chloroform and dried on a rotary evaporator (30 min, 37 °C). The obtained residue was dissolved in 3 mL of phosphate buffer to a final concentration of 5 mmol/L and vortexed until a homogeneous suspension was acquired. The solution was then extruded 21 times by passing the lipids through the 0.2 μm membrane using an Avanti extruder (Avanti Polar Lipids, Alabaster, AL, USA). Liposomes were stored at 4 °C.

### Isothermal calorimetric titration

An isothermal titration calorimeter LV Affinity ITC with gold cells (TA Instruments, New Castle, DE, USA) was applied to evaluate the thermodynamic properties of complexes of HSA or Tf with AuNP14a/b. The protein solution (20 µmol/L in a 1.4275 mL cell) was titrated by adding 50 × 5 µl doses of an 8 mg/mL AuNP solution from the syringe. The time interval between subsequent injections was 1200 s. Isothermal calorimetric measurements were carried out at 25 °C and a stirring speed of 410 rpm. The heat effects of AuNP dilution were determined separately maintaining the same calorimeter parameters. The heat effects of the direct interaction of protein with AuNP were calculated by subtracting the effects of AuNP alone. The binding isotherms describing the heat effects q of the direct interaction of the proteins with nanoparticles as a function of the titrated solution composition expressed as the ratio of AuNP mass per number of protein moles were analyzed for the endpoint of each titration, determined as the cross point of rectilinear fragments in the range of low and high AuNP concentrations.

### Data analysis

All data were calculated from three independent replications and presented as mean ± SD. The normality of the data distributions was determined with the Shapiro-Wilk test. The statistical differences of tested probes to control were assessed using the Kruskal-Wallis test and one-way ANOVA with Dunnett’s multiple comparison test. Mixed-effects analysis was used to compare effects between nanoparticles. All analyses were performed with GraphPad Prism 8 software (GraphPad Software, San Diego, CA, USA).

## Results

### Dynamic light scattering and ζ-potential

**Figure 2:**
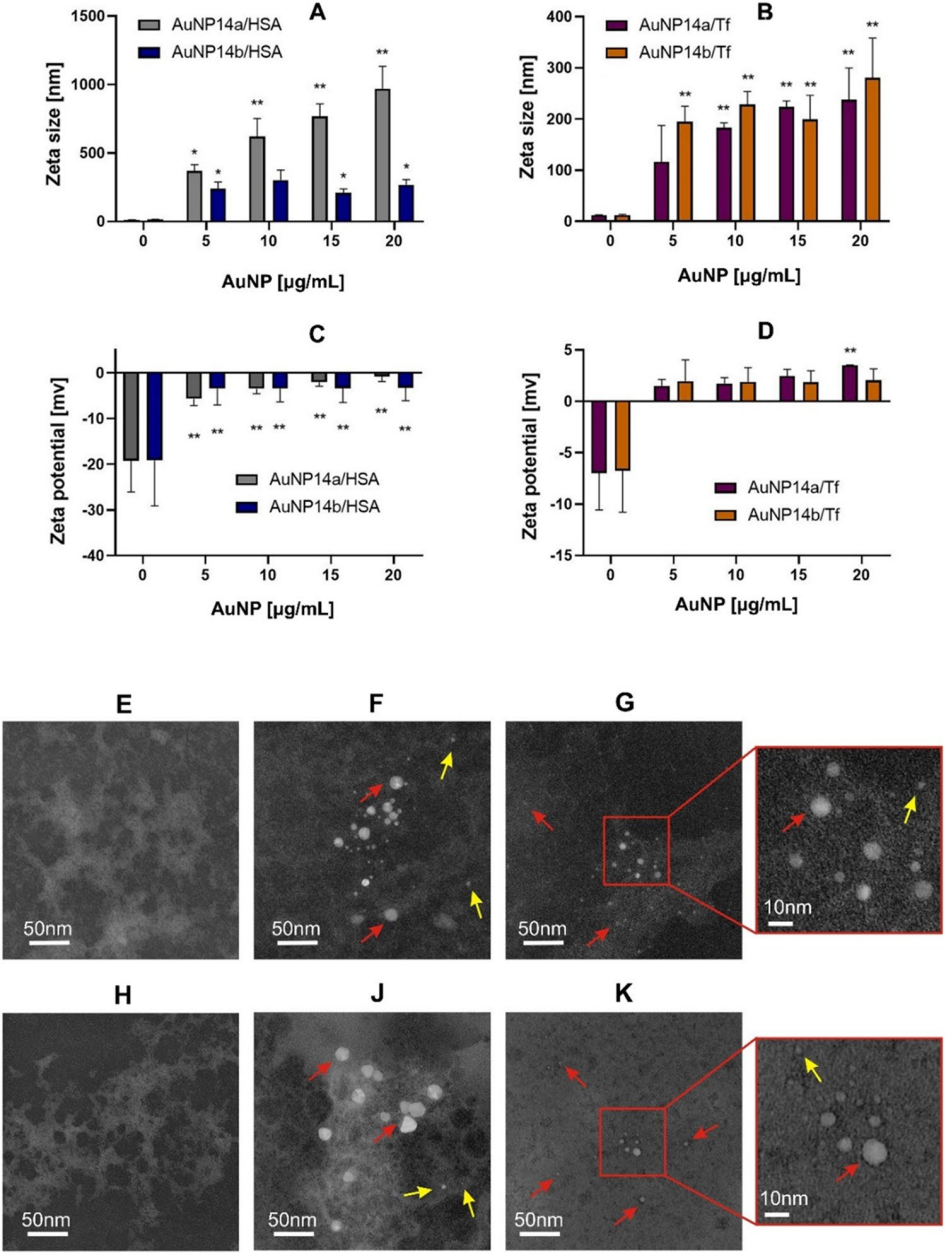
Influence of pegylated gold nanoparticles AuNP1a and AuNP14b on the hydrodynamic diameter expressed as zeta size of human serum albumin (HSA) (**A**) and transferrin (Tf) (**B**), and ζ-potential of HSA (**C**) and Tf (**D**). Graphs present mean values ± SD, * – *p* < 0.05, *** – *p* < 0.001, *n* = 3. Transmission electron microscopy shows the morphology of the proteins in the presence and the absence of AuNP14a/b: HSA (**E**), HSA and AuNP14a (**F**), HSA and AuNP14b (**G**), Tf (**H**) Tf and AuNP14a (**J**) Tf and AuNP14b (K). Red arrows point at large and yellow arrows to small nanoparticle/protein complexes.

Measurements of protein hydrodynamic diameter were made to check whether HSA and Tf form agglomerates with the gold nanoparticles. The mean hydrodynamic diameter of native proteins was 12.03 ± 2.32 nm for HSA and 12.39 ± 0.84 nm for Tf. Upon addition of both nanoparticles hydrodynamic diameter of HSA changed and AuNP14a formed larger aggregates than AuNP14b (Fig. 2A). Increasing concentrations of nanoparticles did not alter the negative charge of the protein (Fig. 2C). Both nanoparticles aggregated with Tf but the hydrodynamic diameter of these conglomerates was smaller than those with HSA (Fig. 2B). ζ-potential of Tf alone was slightly negative and although dosing nanoparticles changed the charge to positive, the difference was not significant (Fig. 2D).

### Transmission electron microscopy

HSA (Fig. 2E) and Tf (Fig. 2H) after addition AuNPs formed aggregates seen as dense clouds around AuNPs (Fig. 2F, G and J, K). AuNPs tended to associate in a protein cluster and agglomerates with larger AuNP14a were captured as more dense electron structures than those with smaller AuNP14b. These observations that the AuNPs interacted with both proteins forming higher-order structures.

### Circular dichroism

**Figure 3:**
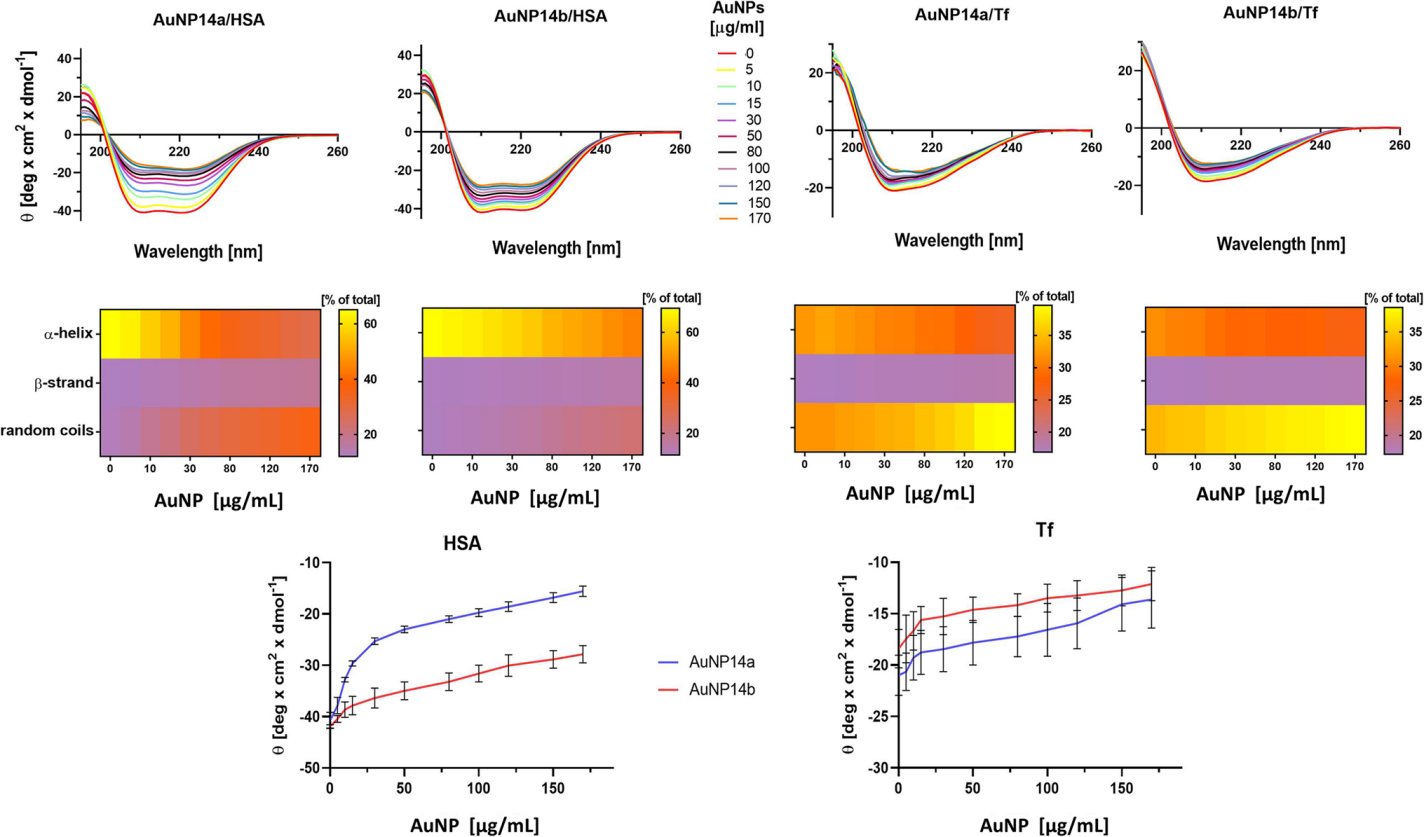
Circular dichroism spectra of human serum albumin (HSA) and transferrin (Tf) incubated with AuNP14a/b nanoparticles expressed by ellipticity (top panel). Heat maps for the contribution of α-helix, β-strand, and random coils conformation to the overall structure of the proteins incubated with the AuNPs (middle panel). Ellipticity of HSA and Tf in the presence of AuNPs at λ 208 nm (bottom panels). Mean ± SD, *n* = 3.

Circular dichroism (CD) measurements provided information about changes in the secondary structure of proteins induced by AuNPs. Both nanoparticles interacted with HSA and Tf changing their spatial structure (Fig. 3). The most pronounced changes were observed for HSA incubated with AuNP14a, which primarily affected the α-helix form of the protein. Both NPs induced similar structural changes in Tf. Data obtained for transferrin was comparable for both tested nanoparticles. Neither AuNP affected the β-strand form of the proteins (Tables S1-S4).

### Fluorescence quenching

Quenching of the fluorescence of tryptophan (Trp), which is the source of intrinsic fluorescence of HSA and Tf, provided information on the binding of AuNPs with these proteins.

Table 1 presents the results of the quenching of fluorescence of HSA and Tf by the AuNP14a/b nanoparticles. It can be seen that AuNP14a quenched the Trp fluorescence more effectively than AuNP14b.

**Table 1:** Quenching of the fluorescence of tryptophan in human serum albumin (HSA) and transferrin (tf) in the presence of gold nanoparticles AuNP14a and AuNP14b.

Protein	AuNPs	[µg/mL]	Fluorescence intensity (mean ± SD)
**HSA**	AuNP14a	0	791.9 ± 48.4
		60	195.3 ± 2.3
	AuNP14b	0	839.1 ± 41.2
		60	465.8 ± 23.6
**Tf**	AuNP14a	0	663.2 ± 26.4
		60	260.8 ± 9.6
	AuNP14b	0	798.5 ± 35
		60	452.7 ± 24.2

### Isothermal titration calorimetry

**Figure 4:**
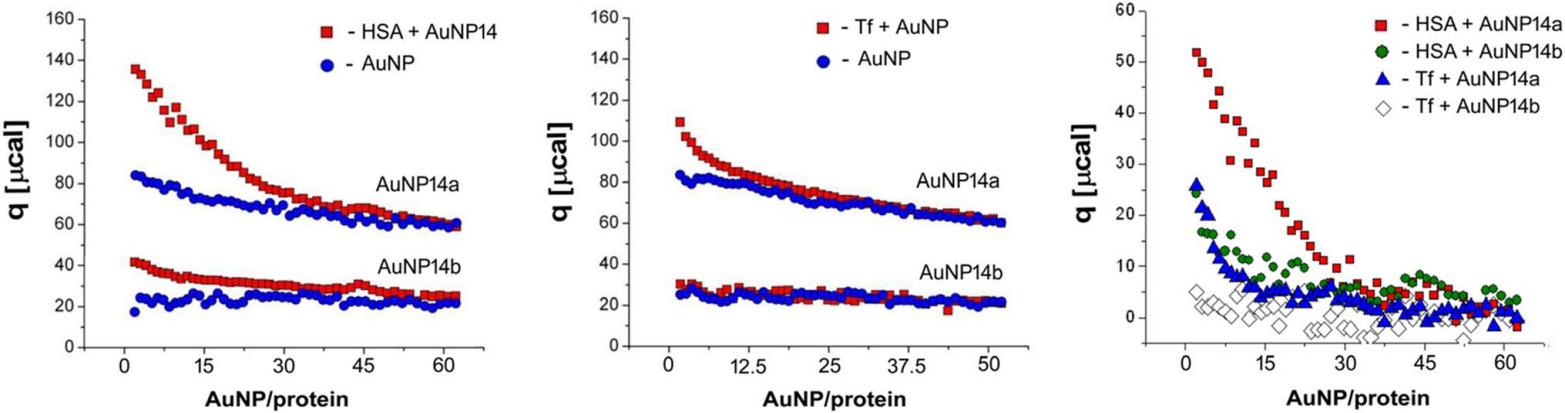
Thermal effects of the interaction between gold nanoparticles AuNP14a/b and human serum albumin (HSA) or transferrin (Tf) evaluated by the heat (q) in isothermal titration calorimetry (left and middle panels). The right panel presents the thermal effects of the direct interaction of the proteins with AuNPs after subtracting the effects of AuNP dilution from their corresponding thermal effects of the titration of the protein solution with AuNP solution.

Isothermal titration calorimetry indicated that both gold nanoparticles interacted with both proteins and all interactions were endothermic (Fig. 4). It can be seen that the most pronounced thermal interaction was for HSA and AuNP14a effect.

### Size, ζ-potential, morphology and fluorescence anisotropy of DMPC liposomes

**Figure 5:**
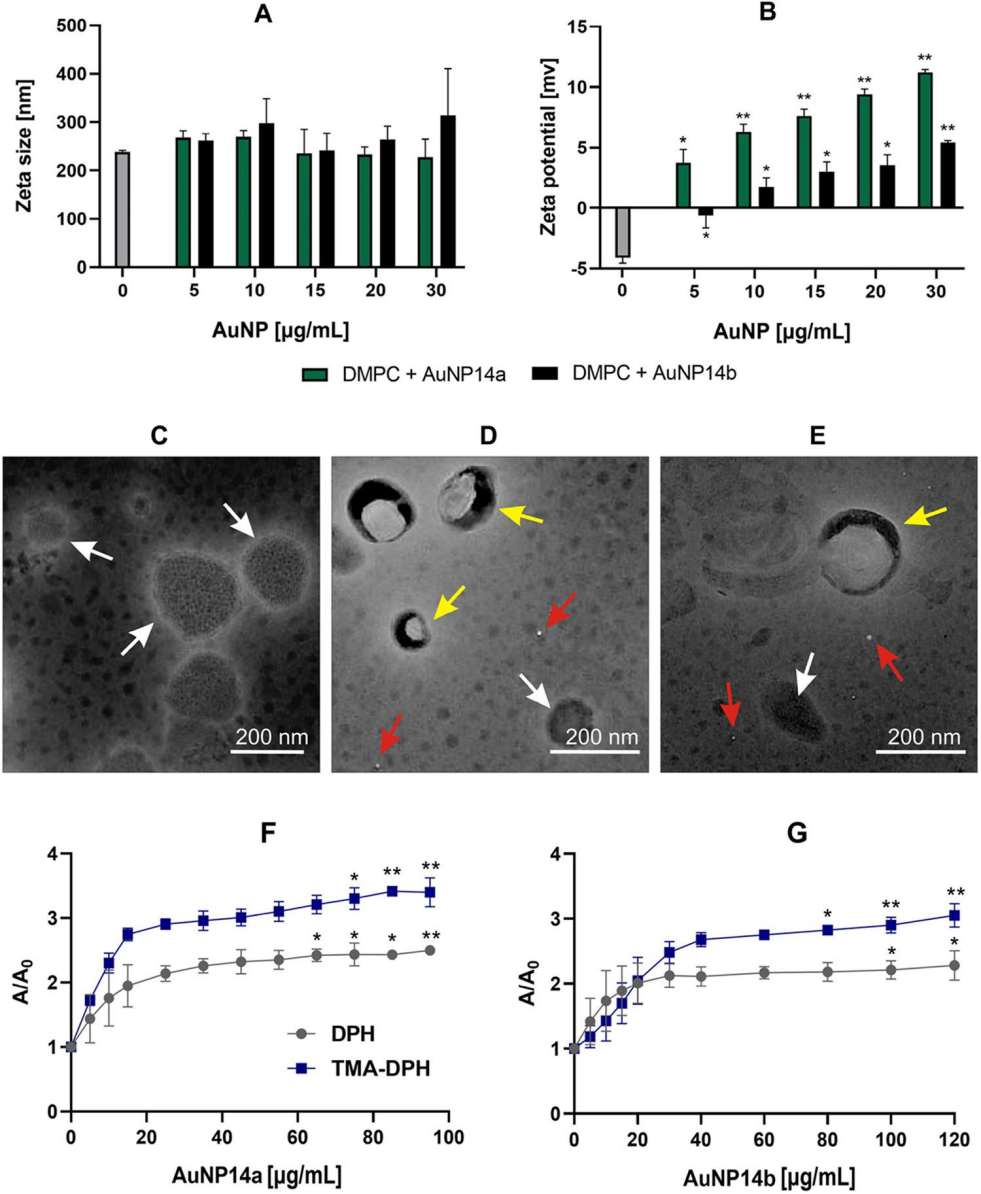
Zeta diameter (**A**) and zeta potential (**B**) of DMPC liposomes in the presence of gold nanoparticles AuNP14a/b. The shape of DMPC liposomes revealed by inverted transmission electron microscopy in the absence (**C**) and the presence of AuNP14a (**D**) and AuNP14b (**E**). White arrows point at free liposomes, red at free gold nanoparticles, and yellow at liposome/nanoparticle complexes. The mean relative fluorescence anisotropy (A/A_0_) of the DPH and TMA-DPH probes added to DMPC liposomes incubated with AuNP14a (**F**) or AuNP14b (**G**). Error bars denote SD, *n* = 3. * – *p* < 0.05; ** – *p* < 0.01 as compared with free liposomes.

The addition of the gold nanoparticles did not change the size of DMPC liposomes as evaluated by dynamic light scattering (Fig. 5A). The ζ-potential of negatively charged DMPC turned positive in most cases after adding AuNPs (Fig. 5B). Without the gold nanoparticles, DMPC liposomes were featured by round, low-density homogenous objects (Fig. 5C). After treatment with AuNP14a/b, the liposomes were characterized by a higher density and more distinct edges. No changes were observed in the liposome size (Fig. 5D and E). Both nanoparticles penetrated the outer and inner parts of the liposome membrane, as evidenced by an increase in anisotropy fluorescence with DPH and TMA-DPH, respectively (Fig. 5F and G).

## Discussion

Gold nanoparticles are widely studied as drug/nucleic acid carriers. Pegylated gold nanoparticles were also investigated as drug carriers. However, many of these studies are limited to the formation of a complex with the cargo and do not consider several factors that may impede the movement of the complex to the target cell. Although it was shown that the surface chemistry is responsible for the interaction of gold nanoparticles with bovine serum albumin, but we extended such study on human albumin and pegylated gold nanoparticles^27^.

In this work, we examined the interaction between proteins, represented by HSA and Tf, and model membranes, represented by DMPC liposomes, with two pegylated gold nanoparticles AuNP14a and AuNp14b considered as potential drug/nucleic acid delivery systems. Therefore, our work provided some data on the possible effects induced by the nanoparticles on their way to the target. In the presence of AuNPs ζ-potential of HSA increased but remained negative. However, AuNPs induced a shift from negative to positive ζ-potential in Tf. These differences may be explained by the differences in the structure of HSA and Tf. Albumin is composed of three homologous domains, Tf has two domains and its main role is the transport of Fe(III) ions, thus it has a high affinity to bind positively charged particles^10,11,28,29^. These features may, at least in part, contribute to a greater susceptibility of HSA to form aggregates than Tf, observed in this work. The isoelectric point (pI) of HSA and Tf is 5.0 and 5.7, respectively. Therefore, the surface charge of both proteins is negative as their pI is below pH of blood in normal conditions (average 7.4) and so both proteins may electrostatically interact with positively charged gold nanoparticles. The optimal zeta potential for drug and nucleic acid delivery by gold nanoparticles depends on several factors, including the cargo properties, the target cells, and their environment, and the physiological environment, generally, nanoparticles with a zeta potential greater than + 25 mV or less than − 25 mV are considered stable in suspension, as the surface charge prevents aggregation^30^. However, for drugs, a slightly negative zeta can help to interact with cell membranes through attractive van der Waals forces, but a positive charge can help in binding the negatively charged nucleic acids. Although all experiments were performed in 10 mmol/L phosphate buffer (pH 7.4), whose pH is not exactly physiological, our previous experience justifies using such buffer as optimal for the biophysical techniques we employed. Transmission electron microscopy showed aggregate formation by HSA and Tf upon the addition of AuNPs. Similar effects were observed and associated with protein corona formation in other studies^31,32^.

We applied different HSA and Trf concentrations using different techniques to obtain an optimal output signal.

Both HSA and Tf displayed specific profiles in the far-UV spectrum observed in circular dichroism measurements. HSA has two negative peaks at 208 nm and 222 nm and Tf has a negative peak at 209 nm and a positive peak at 193 nm^33,34^. Interaction with nanoparticles resulted in changes in these spectra and therefore alteration in the contribution of α-helix and random coils conformation in the overall structure of both proteins. We did not perform functional studies for HSA and Tf in the presence or absence of gold nanoparticles. Therefore, it is not possible in this work to determine how the binding of these proteins by gold nanoparticles influenced their functionality. However, as the higher-order structure of a protein is a major determinant of its functionality, some conclusions can be drawn from the CD experiments. Changes in chirality and loss of the α-helix structure of protein lead to its denaturation. This is a common effect observed in protein-nanoparticle interaction^34,35^. Circular dichroism spectra analysis and alteration in secondary structures help to understand how the functionality is changing and whether the process of denaturation can be reversible. The greatest loss of α-helix was observed in HSA after AuNP14a addition (Tables S1-S4). Transferrin was less susceptible to secondary structure loss upon AuNPs addition. In general, Tf was found to be less susceptible to spectral and structural changes. Increasing concentrations of gold nanoparticles increased the random coil fraction.

Quenching of intrinsic protein fluorescence by nanoparticles is usually exploited to determine the kinetics of binding but here we used it to show how AuNPs affected protein conformation. Tryptophan is present in HSA and Tf in different amounts and accessibility. HSA has one Trp in the hydrophobic pocket of the IIA domain, while Tf has eight Trp residues among which 3 are in the N-lobe and 5 in the C-lobe^36,37^. Conformational changes can be evidenced by quenching spectrum shifts toward smaller wavelengths (blueshifts) or higher wavelengths (redshift)^38^. In our study, we observed a slight redshift for HSA with both AuNPs. Redshift indicates that Trp became more exposed during AuNPs titration and therefore HSA structure may become loose. Tf did not display any shift and strong fluorescence decline at the same time as presented in Table 1. The greater amount of Trp and its worse accessibility may be the reason for the smaller sensitivity of Tf to AuNPs^39^. Tested nanoparticles do not display fluorescence in measured wavelengths. Isothermal titration calorimetry showed that all protein-AuNP interactions were endothermic, and the strongest endothermic effect was observed for AuNP14a with HSA. This result provides information about the high affinity of AuNP14a to albumin. The reaction of transferrin with AuNP14b showed “no end point” of titration and this outcome could be interpreted as the low affinity of nanoparticle to this protein^39^.

Measurements of the zeta diameter and zeta potential of liposomes complexed with AuNPs showed that DMPC liposomes did not change their zeta size in the presence of AuNPs, but they changed their zeta potential from negative to positive. These results indicate that AuNPs interacted with lipid membranes, but they did not disrupt them. TEM images show that liposomes remained spherical after treatment with AuNPs and due to the density alterations, nanoparticles might penetrate liposomes through the lipid bilayer. The size of DMPC complexed with AuNPs differed between DLS measurements and TEM micrographs, but this phenomenon can be explained by differences in sample preparation, high vacuum effect causing sample collapsing into the desiccated layer in TEM or DLS property to hinder smaller particles by larger ones^40–42^.

Membrane fluidity can be directly related to its permeability^43^. Measurements of fluorescence anisotropy help to assess bilayer microviscosity that is inversely related to fluidity^44^. Our data suggest that both nanoparticles increased the microviscosity of the inner and outer parts of the membrane, but AuNP14a caused significant changes at smaller concentrations than AuNP14b. These results lead to the conclusion that AuNP14a and AuNP14b interact with a hydrophilic head and can penetrate the hydrophobic tails of phospholipids. This statement is consistent with ζ-potential and TEM results showing that the nanoparticles change the charge of the outer part of the membrane and diffuse through the bilayer of the liposome. Electrostatic interactions of gold atoms with hydrophilic heads of the membrane were shown suggesting that they might be independent of the size of gold nanoparticles^45,46^.

This work showed that the AuNP14a/b nanoparticles may penetrate a lipid membrane, suggesting that they may reach the cytoplasm of a live cell. Therefore, they may be considered a potential drug/nucleic acid carrier, but further studies are needed to determine binding constant and the number of binding sites for the drug or nucleic acid of interest^47^.

It is a limitation of our work that we did not check whether the system reached equilibrium and we did not investigate the kinetics of the interactions and the influence of protein precipitation on fluorescence intensity.

We performed a series of in vitro experiments, but we intended to obtain some data that could be related to in vivo conditions. The general conclusion is that if the gold nanoparticles we used were applied as drug/nucleic acid carriers, they would interact with HSA and Tf. However, it is not possible to determine how strong such interactions would be as it is not possible to determine or even approximate the binding constants for complexes of the nanoparticles with other blood proteins.

## Conclusions

Pegylated gold nanoparticles can interact with human serum albumin and transferrin changing their properties, including their secondary structure. Further studies are needed to determine whether these changes are associated with the changes in the functionality of HSA and Tf and if they can be harmful to the cell/organism. These nanoparticles may penetrate DMPC liposomes suggesting that they may also diffuse through natural membranes and consequently may be considered as drug/nucleic acid carriers in the therapy.

## Supplementary Information

Supplementary Material 1.

## Data Availability

The data that support the findings of this study are available from the corresponding authors: Elżbieta Okła, Tel.:+48 42 635 43 80, e-mail: elzbieta.okla@biol.uni.lodz.plMaksim Ionov, Tel.:+48 42 635 43 80, e-mail: maksim.ionov@biol.uni.lodz.pl.

## Abbreviations

AuNPs: gold nanoparticles
Tf: transferrin
siRNA: small interfering RNA
BBB: the blood-brain barrier
DMPC: 1,2-dimyristoyl-sn-glycero-3-phosphocholine
DLS: Dynamic light scattering
RI: refractive index
CD: Circular dichroism
DPH: (1,6-diphenylhexatriene) and its hydrophilic derivative
TMA-DPH: (1-(4-Trimethylammoniophenyl)-6-phenyl-1,3,5-hexatriene p-toluenesulfonate)
TEM: transmission electron microscopy
ITC: isothermal titration calorimetry
HSA: human serum albumin
PEG: polyethylene glycol
CD: circular dichroism
