# Supplementary material for: Pegylated gold nanoparticles interact with lipid bilayer and human serum albumin and transferrin
Source: Sci Rep. 2024 Oct 18;14:24408. doi: 10.1038/s41598-024-74898-0 (PMC11487075; doi:10.1038/s41598-024-74898-0)
Supplement: Supplementary file 1 — Supplementary Material 1. [file 41598_2024_74898_MOESM1_ESM.docx]

**Suplementarny Information**

**Pegylated gold nanoparticles as potential drug/nucleic acid carriers: interaction with lipid bilayer and human serum albumin and transferrin**

Elżbieta Okła ^1,2^*, Sylwia Michlewska ^3^, Adam Buczkowski ^4^, Serafin Zawadzki ^1,5^, Katarzyna Miłowska ^1^, Javier Sánchez-Nieves ^6,7^, Rafael Gómez ^6,7^, Francisco Javier de la Mata ^6,7^, Maria Bryszewska ^1^, Janusz Blasiak ^8^, Maksim Ionov ^1,8^*

*^1^ University of Lodz, Faculty of Biology and Environmental Protection, Department of General Biophysics, Pomorska 141/143, 90–236 Lodz, Poland.*

*^2^ University of Lodz Doctoral School of exact and natural sciences , 21/23 Matejki St., 90-237, Lodz, Poland.*

*^3^ University of Lodz, Faculty of Biology and Environmental Protection, Laboratory of Microscopic Imaging and Specialized Biological Techniques, Banacha 12/16, 90–237 Lodz, Poland.*

*^4^ University of Lodz, Faculty of Chemistry, Department of Physical Chemistry, Division of Biophysical Chemistry, Pomorska 165, 90-236 Lodz, Poland.*

*^5^ BioMedChem Doctoral School of the University of Lodz and Lodz Institutes of the Polish Academy of Sciences, 21/23 Matejki St., 90‑237 Lodz, Poland.*

*^6^ Universidad de Alcalá Department of Organic and Inorganic Chemistry, and Research Institute in Chemistry "Andrés M. del Río" (IQAR), Spain and Instituto Ramon y Cajal de Investigacion Sanitaria, IRYCIS, Colmenar Viejo Road, Km 9, 100, 28034 Madrid, Spain.*

*^7^ Networking Research Center on Bioengineering, Biomaterials and Nanomedicine (CIBER-BBN), Spain.*

*^8^ Mazovian Academy in Plock, Collegium Medicum, Faculty of Medicine, Pl. Dabrowskiego 2, 09-402 Plock, Poland.*

**Table S1.** Changes in secondary structures of **HSA** upon **AuNP14a** administration. Data shows mean values ± SD of three independent repeats.

| AuNP14a [µg/ml] | α-helix [%] ± SD | β-strands [%] ± SD | random coil [%] ± SD |
| --- | --- | --- | --- |
| 0 | 65.10 ± 5.11 | 12.07 ± 0.64 | 12.77 ± 2.03 |
| 5 | 62.63 ± 0.84 | 12.37 ± 0.15 | 14.63 ± 0.32 |
| 10 | 56.70 ± 0.10 | 13.10 ± 0.00 | 18.37 ± 0.12 |
| 15 | 52.20 ± 0.26 | 13.70 ± 0.00 | 20.90 ± 0.44 |
| 30 | 44.90 ± 0.96 | 14.77 ± 0.15 | 25.07 ± 0.55 |
| 50 | 40.07 ± 0.95 | 15.50 ± 0.10 | 27.57 ± 0.83 |
| 80 | 35.77 ± 0.57 | 16.23 ± 0.12 | 29.83 ± 0.68 |
| 100 | 33.63 ± 0.42 | 16.60 ± 0.10 | 31.37 ± 0.72 |
| 120 | 32.27 ± 0.96 | 16.87 ± 0.15 | 32.23 ± 0.85 |
| 150 | 29.67 ± 0.40 | 17.30 ± 0.00 | 34.67 ± 0.97 |
| 170 | 28.07 ± 0.40 | 17.60 ± 0.10 | 36.03 ± 0.97 |

**Table S2.** Changes in secondary structures of **HSA** upon **AuNP14b** administration. Data shows mean values ± SD of three independent repeats.

| AuNP14b [µg/ml] | α-helix [%] ± SD | β-strands [%] ± SD | random coil [%] ± SD |
| --- | --- | --- | --- |
| 0 | 69.45 ± 0.64 | 11.45 ± 0.07 | 11.40 ± 0.14 |
| 5 | 67.53 ± 0.49 | 11.77 ± 0.06 | 12.20 ± 0.17 |
| 10 | 66.33 ± 1.75 | 11.90 ± 0.26 | 13.03 ± 0.81 |
| 15 | 64.27 ± 3.16 | 12.23 ± 0.45 | 13.77 ± 1.30 |
| 30 | 61.97 ± 2.80 | 12.50 ± 0.35 | 14.87 ± 1.38 |
| 50 | 59.47 ± 2.29 | 12.87 ± 0.32 | 15.90 ± 1.13 |
| 80 | 55.90 ± 2.17 | 13.37 ± 0.32 | 17.63 ± 1.15 |
| 100 | 53.50 ± 1.93 | 13.67 ± 0.32 | 18.97 ± 1.02 |
| 120 | 51.63 ± 2.42 | 13.90 ± 0.35 | 20.40 ± 1.42 |
| 150 | 48.47 ± 1.78 | 14.37 ± 0.23 | 21.67 ± 1.10 |
| 170 | 46.43 ± 1.79 | 14.67 ± 0.23 | 22.80 ± 1.22 |

**Table S3.** Changes in secondary structures of **Tf** upon **AuNP14a** administration. Data shows mean values ± SD of three independent repeats.

| AuNP14a [µg/ml] | α-helix [%] ± SD | β-strands [%] ± SD | random coil [%] ± SD |
| --- | --- | --- | --- |
| 0 | 32.27 ± 2.74 | 17.07 ± 0.49 | 31.90 ± 1.90 |
| 5 | 33.00 ± 1.35 | 16.90 ± 0.20 | 31.90 ± 0.96 |
| 10 | 32.23 ± 2.30 | 17.03 ± 0.45 | 32.67 ± 1.83 |
| 15 | 31.50 ± 2.09 | 17.20 ± 0.35 | 33.07 ± 1.72 |
| 30 | 30.70 ± 1.91 | 17.33 ± 0.38 | 33.73 ± 1.65 |
| 50 | 30.43 ± 0.50 | 17.37 ± 0.12 | 34.13 ± 1.05 |
| 80 | 29.60 ± 0.72 | 17.50 ± 0.20 | 35.10 ± 0.66 |
| 100 | 29.50 ± 1.73 | 17.47 ± 0.35 | 35.93 ± 1.65 |
| 120 | 28.17 ± 2.50 | 17.70 ± 0.53 | 37.07 ± 1.40 |
| 150 | 26.87 ± 2.70 | 17.87 ± 0.70 | 39.17 ± 0.51 |
| 170 | 26.27 ± 1.95 | 18.00 ± 0.50 | 39.63 ± 1.14 |

**Table S4.** Changes in secondary structures of **Tf** upon **AuNP14b** administration. Data shows mean values ± SD of three independent repeats.

| AuNP14b [µg/ml] | α-helix [%] ± SD | β-strands [%] ± SD | random coil [%] ± SD |
| --- | --- | --- | --- |
| 0 | 31.00 ± 1.15 | 17.27 ± 0.21 | 33.70 ± 1.25 |
| 5 | 30.07 ± 2.07 | 17.43 ± 0.40 | 34.50 ± 1.75 |
| 10 | 30.03 ± 1.88 | 17.40 ± 0.35 | 34.70 ± 1.47 |
| 15 | 28.87 ± 1.17 | 17.67 ± 0.23 | 35.53 ± 1.01 |
| 30 | 28.23 ± 1.00 | 17.80 ± 0.20 | 36.17 ± 1.36 |
| 50 | 28.33 ± 0.87 | 17.73 ± 0.21 | 36.40 ± 1.08 |
| 80 | 27.77 ± 1.00 | 17.83 ± 0.21 | 37.00 ± 1.06 |
| 100 | 27.80 ± 1.49 | 17.83 ± 0.29 | 37.40 ± 1.37 |
| 120 | 28.07 ± 1.63 | 17.77 ± 0.32 | 37.53 ± 1.31 |
| 150 | 27.07 ± 1.15 | 17.93 ± 0.21 | 38.13 ± 1.23 |
| 170 | 27.10 ± 0.80 | 17.93 ± 0.12 | 38.53 ± 1.36 |
